# In Memoriam—Dr. Wm. Pepper

**Published:** 1898-09

**Authors:** 


### IN MEMORIAM—DR. WM. PEPPER.

On Friday, July the 29th, at 12 noon, there appeared on the bulletin board of the *Press* office at Seventh and Chestnut streets, Philadelphia, in large letters, this announcement: "Dr. William Pepper died suddenly last night in California." The crowds that surged up and down that thoroughfare soon spread this sad news over the whole city. Could it be true? Was there not some possible mistake: and Philadelphia still could welcome the return of her son, who was not only honored by his fellow-citizens, but whose far-famed reputation was universal? Alas, the notice was only too true! At a later hour the editions of the evening journals gave an account of the last hours of one of the greatest men of the century.

Dr. Pepper's mind power was extraordinary. He not only excelled in his chosen profession, but he was gifted with great powers of persuasion by which he influenced alike the City Councils of Philadelphia and the Legislature of Pennsylvania, the one to the donation of large and valuable tracts of land in the city, and the other of large sums of money to the one great object, the advancement of the University of Pennsylvania. Wealthy citizens were in like manner induced to open their purses to this same beloved object, and even in commercial life he stirred the citizens to a keener appreciation of their own interests by inducing them to found a free commercial museum where exhibits are shown of the products of all nations.

He was a greater man than any one of his contemporaries. Such men as Leidy and Cope were great men, but their greatness was confined to some one specialty, but Dr. Pepper was great in the versatility of his mind. His wonderful executive ability made him successful in everything he undertook, and his amiable disposition played an important part in contributing to his success.

To every person with whom he talked he showed a marked affability. No matter what that person's station in life might be, Dr. Pepper made him feel that he had a friend who regarded his interests.

He was a natural orator. Before the classes in the college, before legislative committees, in the meetings of numerous societies to which he belonged, and before conventions of citizens assembled for various public-spirited purposes his style was incisive, graceful, persuasive, instructive, and most eloquent.

The result of his energy as Provost was that the University has acquired in land, buildings, and money since 1891, not less than four millions of dollars. From three buildings, the University has expanded to more than thirty buildings, covering an area of fifty-two acres, and the value of all this property has risen from less than half a million dollars to probably ten millions. Its reputation in like proportion has increased from a merely local value to a world-wide celebrity, rivaling Harvard and Yale colleges in distinction.

Dr. Pepper founded the *Medical Times* and was its editor in 1870 and 1871. He was medical director of the Centennial Exhibition at Philadelphia in 1876. He was the principal founder of the Pennsylvania Museum and School of Industrial Art. He was a member of the College of Physicians of Philadelphia county; of the American Philosophical Society; of the Pathological Society of Philadelphia; of the American Academy of the Natural Sciences, and its President from 1873 to 1876. He was president of the Pan-American Congress in Washington in 1893. He was given the degree of LL. D. by Princeton College in 1888, and was the editor of *The System of Medicine by American Authors*, besides being the author of numerous other medical works.

He founded the Museum of Archæology and Palæontology of the University, which bids fair to become one of the greatest in this country.

Thus his versatility before mentioned is made apparent, and in the language of an editor who was his intimate friend, "His sad and untimely end leaves much that he planned and would have accomplished, incomplete."

He was a born diplomat, a great financier, a distinguished medical man, an accurate and sagacious diagnostician, and a versatile scientific man. There is none to take his place.

Some surprise may be elicited that this journal should give up so much of its space to the obituary notice of a physician who, however distinguished in his career, was nevertheless not a homœopathist, and was generally supposed to be hostile to Homœopathy.

This supposition was, however, erroneous. Dr. Pepper, in the latter years of his life, became impressed with the idea that there was a great underlying truth in Homœopathy. He never advocated it; he never practiced it, yet he insensibly fell into the modes of thought that would suggest a knowledge of Homœopathy.

In his office he has been overheard to advise a patient to cease taking strong medicine and to remain away from him until the patient's system had sufficiently freed itself from medicinal influence to give him an opportunity to observe its condition unobscured by drug action.

Once, when Dr. Pepper was paying a visit to the editor of this journal, the conversation turned upon Homœopathy. The latter took occasion to regret the antagonistic tone of many of Hahnemann's writings, as tending to repel those who might otherwise read them. To his surprise Dr. Pepper promptly defended Hahnemann, reminding the editor of the severity of the persecutions of the master, and that he had been driven to this antagonistic style by these persecutions.

Twenty years ago Dr. Pepper did write a pamphlet against Homœopathy which aroused the strong indignation of Dr. Lippe. Dr. Lippe replied in another pamphlet in his usual vigorous style. These two pamphlets are unfortunately missing, else they would both now be published in these pages.

In later years Dr. Pepper had experienced a change of opinion concerning this great principle of treatment, as the foregoing anecdote of conversation plainly shows. Indeed, he complained on that occasion that his great difficulty with Homœopathy was the utter contempt for and nullification of its principles by its professed advocates and partisans.

He was so broad-minded that he would willingly have admitted the teaching of Homœopathy into the University of Pennsylvania, had it not been for the opposition of his fellow-practitioners who had not his liberal intelligence and keen penetration of the future.

For this favorable attitude toward Homœopathy, for his liberal desire to establish it in its proper place as a part of a University education we cordially commend to the homœopathic profession the memory of Dr. William Pepper.

H. A. P. J.

